# Cellular therapies in trauma and critical care medicine: Looking towards the future

**DOI:** 10.1371/journal.pmed.1002343

**Published:** 2017-07-11

**Authors:** Shibani Pati, Todd E. Rasmussen

**Affiliations:** 1 Department of Laboratory Medicine, University of California San Francisco, San Francisco, California, United States of America; 2 Blood Systems Research Institute, San Francisco, California, United States of America; 3 F. Edward Hebert School of Medicine, Uniformed Services University of Health Sciences, Bethesda, Maryland, United States of America

## Abstract

Shibani Pati and Todd Rasmussen summarize progress in preclinical research on cellular therapeutics for traumatic injury and its sequelae and discuss prospects for clinical translation.

## Background

Trauma is the leading cause of death for individuals between the ages of 1–44 worldwide.[1] In recent years, improved methods to stop bleeding and optimally resuscitate patients have increased the overall survival and decreased the morbidity associated with severe hemorrhage and trauma. [2–4] There are, however, few therapeutic interventions that mitigate intermediate and long-term outcomes in patients who survive the initial injury, a population whose numbers have increased with recent successful measures at improving initial survival from combat-related injury. With few therapeutic options beyond supportive care, trauma-related mortality and morbidity is an area with unlimited scope for advancement.

A novel, emerging area of investigation that has generated considerable interest is the potential use of cellular therapies (CT) to prevent secondary injury and promote repair of injured tissue in trauma.[5] Blood transfusion, having been used since the 19th century, is in fact the first cell therapy to be utilized in bleeding trauma patients. In the United States Civil War (1861 to 1865), hemorrhage caused three-fourths of combat-related deaths, [6] and the first blood transfusion recorded in this setting was conducted by surgeon Edwin Bentley to treat a soldier with a gunshot wound who required a leg amputation.[7]

In recent years, largely spurred by interest and investment from the US military’s trauma injury research program, the field of cellular therapeutics and regenerative medicine has grown rapidly. CT have been investigated preclinically and clinically for applications in trauma.[5] Although the field is still in its early stages of development, animal and human studies demonstrate the promise of CT for trauma-induced conditions, such as traumatic brain injury (TBI), spinal cord injury (SCI), organ failure (Acute Respiratory Distress Syndrome [ARDS], Acute Kidney Injury [AKI]), orthopedic trauma, burns, as well as a number of adverse conditions in the severely injured extremity, including soft tissue damage and ischemia reperfusion injury.[5,8–24]

## Types of CT and mechanisms of action

A multitude of cell types derived from a variety of tissues are currently under preclinical and clinical investigation for applications in trauma. CT fall into 2 main categories of cell types: adult multipotent cells and pluripotent embryonic stem cells (ESCs). Induced pluripotent stem cells (IPSCs) are a third cell group that are derived from de-differentiated adult cells.[5] Adult multipotent cells, such as mesenchymal stem cells (MSCs), multipotent adult progenitor cells (MAPCs), hematopoietic stem cells (HSCs), and bone marrow mononuclear cells (BMMNCs), have the capacity to generate a limited number of terminally differentiated cell types.[5] Currently, there are over 500 clinical trials for MSCs listed on ClinicalTrials.Gov, investigating their therapeutic potential in a variety of clinical applications and demonstrating an excellent track record of safety. MSCs are likely to be the first main cell type that will pass regulatory approval and proceed to commercialization for clinical use in the next few years, both in the US and worldwide. Pluripotent stem cells such as ESCs or IPSCs have the capacity for continuous self-renewal and can differentiate into any cell type in the body.[25] Clinical use of these cells typically involves specialized cells that have been derived from ESCs and grown under defined conditions to produce a particular cell type of interest.[26]

Mechanistically, cell-based therapies have been shown to improve outcomes in preclinical studies of trauma-related conditions characterized by uncontrolled inflammation, vascular compromise, and aberrant coagulation: a pathophysiological triad known as the endotheliopathy of trauma (EOT). CT mechanisms of action include the following: (1) producing soluble factors that regulate the EOT (i.e., growth factors, cytokines, chemokines, microvesicles, exosomes, and mitochondria) through anti-inflammatory and cell-protective effects [27], (2) replacing cells that are lost by differentiating and integrating into the damaged tissue microenvironment, and (3) stimulating regeneration and repair of endogenous injured tissue. Multipotent cells are optimally oriented towards prevention and repair of injured tissue, and pluripotent stem cells are ideally suited for cell replacement of lost and injured tissue. In contrast to small molecule therapies, CT have the potential to modulate pleotropic therapeutic targets and thereby address the heterogeneity of disease present in the trauma patient.[22]

## Target patient population for CT in trauma

Identifying which cohort of trauma patients would most likely benefit from CT and which endpoints are best matched to the mechanism of action defined for the particular cell type under investigation is critical to developing successful clinical trials. In terms of mortality, there are 3 main cohorts of patients defined by time postinjury. In the first 2 hours after injury, approximately 25% of the deaths that occur are secondary to hemorrhage.[2] These patients can be saved by surgery, hemostatic interventions, and optimal resuscitation paradigms and will not likely benefit from CT. During the second period, between 8 hours and 3 days after injury, most of the deaths are the result of severe TBI [28]. There is likely a small population of patients in this group that may benefit from a cell-based therapy, i.e., for the control of cerebral edema and to limit increases in intracranial pressure (ICP).[2] After 3 days postinjury, the remaining 25% of deaths occur at a low and continuous rate, extending up to 30 days or more postinjury.[5] EOT is a key factor in these deaths.[29,30] EOT contributes to the development of trauma-induced multi-organ failure (MOF), sepsis, ARDS, venous thromboembolic disease (VTE), and AKI. Aside from supportive care, there are few treatment options for these clinical endpoints, and based on defined mechanisms of action, these endpoints are likely targets for amelioration using CT.

## Investigation of CT in trauma

Preclinical studies in animal models have provided insight on the potential of various CT and their mechanisms of action, but no single model can fully recapitulate the complex heterogeneity of the disease. While research is advancing in multiple areas of clinical investigation, including orthopedic trauma and wound healing [5], we focus here on the areas of neurotrauma (TBI and SCI) and organ failure (ARDS).

Neurotrauma remains a significant public health concern in both civilian and military populations worldwide.[31] Although CT have shown promise in treating applications in neurotrauma such as TBI and SCI, translatable CT are still in early development. Cell types investigated in TBI preclinical models include BMMNCs, MSCs, and MAPCs. All 3 of these cells have demonstrated significant therapeutic effects on regulating blood–brain barrier permeability, neuroinflammation, neuroprotection, and neurocognitive outcomes.[19,32–34] Building on supportive preclinical data, a phase 1 clinical trial investigated intravenous administration of autologous BMMNCs for the treatment of severe TBI in children, primarily to evaluate treatment safety. [35] The trial demonstrated the feasibility, safety, and some evidence of efficacy for BMMNCs in TBI.[35] Phase 2 studies are now ongoing in both children and adults with TBI, with a focus on unique trial design and assessment of outcome measures related to structural imaging of the brains of injured and treated patients.[21,35,36]

SCI involves a myriad of adverse processes that are potential therapeutic targets for CT. These include increased oxidative stress, inflammation, demyelination, blood–spinal cord barrier permeability, increasing cavity lesion size, glial scarring, and decreased neural connectivity.[23,37] Cell types investigated in SCI preclinical models of injury include ESCs, MSCs, neural stem cells (NSCs), olfactory ensheathing glia (OEG), oligodendrocyte progenitor cells (OPCs), Schwann cells (SC), and activated macrophages[38–40]. There is currently a phase 1/2a trial for human ESC–derived OPCs, which are transplanted weeks after injury for American Spinal Injury Association (ASIA)-A patients with complete cervical SCI. These are patients who lack motor and sensory function in S4–S5 levels. Promising safety outcomes and some initial reports of efficacy have been recently reported.[26,41]

In the category of organ failure, CT using bone marrow–derived MSCs have been investigated extensively for ARDS. [16,24,42] Preclinical research has demonstrated the effectiveness of MSCs in decreasing pulmonary vascular permeability, inflammation, and lung edema while increasing macrophage phagocytosis of bacteria.[22,43,44] Many of these effects have been shown to be mediated by soluble factors that are secreted by the MSCs postinfusion.[22,44] MSCs have been investigated in a multicenter, phase 1/2a, randomized, placebo-controlled trial of nontrauma-related ARDS. This trial reported safety in dose escalation studies; currently, the Phase 2a trial is complete, but results have yet to be reported.[42] Recently, the Department of Defense (DOD) has funded a multicenter, randomized, placebo-controlled trial starting in late 2017 in the US for trauma-induced ARDS. This study will be the first to clinically investigate the use of MSCs for ARDS in trauma patients. Overall, definitive evidence on the clinical safety and efficacy of CT in trauma is not yet available, but with the completion of many of these current and planned trials, the next 5 years should bring answers to many questions.

## Current challenges for CT in trauma

Challenges remain with regard to the translation of CT into practice. For example, the assessment of which cell types and cell sources are most likely to improve outcomes of severe injury is still in its early stages. Additionally, research and development is needed to characterize different processing methods, doses, route, and timing of administration. Advances in each of these categories are needed to inform the effective design and conduct of clinical studies. Another critical area of development is the reliable production of clinical-grade cells in sufficient quantities for trials and eventual licensure. A fact underscoring the relatively nascent state of clinical work in this area is the absence of a Food and Drug Administration (FDA)- or European Medicines Agency (EMA)-approved, commercially-derived CT for trauma or critical care medicine, but even if FDA or EMA clearance were to be obtained for any of the MSC-based therapies, current sources of clinical-grade cells would probably not be sufficient to meet demand. Optimal methods to support large-scale cell expansion and processing of the various cell products is still in development. Each cell type requires its own specific conditions for optimal growth, which may also be disease application–dependent.

Regulatory challenges also exist for CT. The regulatory roadmap is still under development at European and US regulatory agencies.[45–48] Regulatory concerns specific to trauma in the US include consent issues pertaining to exception from informed consent (EFIC) under the US Code of Federal Regulations Title 21 Part 50, which would likely be needed for clinical trials in trauma in which CT would have to be administered at early time points when direct consent of the patient is not possible. Informed consent issues comprise one of several challenges pertaining to optimal clinical trial design in trauma. For example, a lack of success in TBI trials[49–51] may be attributed to suboptimal trial design compounded by the heterogeneity of the disease and the need to identify modifiable outcome measures.[52] Addressing similar issues will be key to the clinical advancement of CT in trauma.

Finally, adequate and reliable sources of medical research funding are required to support preclinical and human subject research. In the US, some states have started agencies, such as the California Institute of Regenerative Medicine (CIRM), that aim to support regenerative medicine research efforts. The National Institutes of Health (NIH) and the DOD have also embarked on specific endeavors to support foundational, applied, and clinical study of CT for the treatment of trauma-related conditions. Although injury is a leading cause of death and disability in the US, research funding for this condition and investigation of therapies focused on mitigating its impact are low in comparison to the support aimed at cancer and infectious and cardiovascular diseases [3].

### Looking towards the future for CT in trauma

Increasing awareness of the promise of CT, and the impossibility of developing a single drug to address the heterogeneous states of trauma and injury, led the DOD to initiate a “state of the science” meeting in 2015 [5]. Subsequent publications on the topic led to discussion, planning, and programming of a portion of the DOD's trauma and injury research funding towards this topic. These focused efforts resulted in multiple research awards to civilian and DOD research groups, supporting both clinical and preclinical work; the hope is that new knowledge and materiel products stemming from this investment will advance the field and improve survival and recovery of those injured in military and civilian settings. CT have demonstrated a unique and exciting potential to limit the sequelae of severe injury and, in doing so, improve survival and recovery. Committing to this topic area and working in a coordinated fashion, civilian and military researchers, clinicians, and funding organizations can realize this potential.

## Abbreviations

AKI: Acute Kidney Injury
ARDS: Acute Respiratory Distress Syndrome
ASIA: American Spinal Injury Association
BMMNC: Bone Marrow Mononuclear Cell
CIRM: California Institute of Regenerative Medicine
CT: Cellular Therapies
DOD: Department of Defense
EOT: Endotheliopathy of Trauma
ESC: Embryonic Stem Cell
EMA: European Medicines Agency
EFIC: Exception from Informed Consent
FDA: Food and Drug Administration
HSC: Hematopoietic Stem Cell
ICP: Intracranial Pressure
IPSC: Induced Pluripotent Stem Cell
MOF: Multi-Organ Failure
MSC: Mesenchymal Stem Cell
MAPC: Multipotent Adult Progenitor Cell
NIH: National Institutes of Health
NSC: Neural Stem Cell
OEG: Olfactory Ensheathing Glia
OPC: Oligodendrocyte Progenitor Cells
SC: Schwann Cells
SCI: Spinal Cord Injury
TBI: Traumatic Brain Injury
VTE: Venous Thromboembolic Disease
